# Clinical and Laboratory Findings in Children with Hashimoto’s Thyroiditis

**DOI:** 10.5152/eurasianjmed.2024.24541

**Published:** 2024-10-01

**Authors:** Ayşe Ozden, Hakan Doneray

**Affiliations:** Department of Pediatric Endocrinology, Ataturk University Faculty of Medicine, Erzurum, Türkiye

**Keywords:** Autoimmune thyroiditis,, Hashimoto’s disease,, hypothyroidism

## Abstract

**Background:**

Hashimoto’s thyroiditis (HT) is the most common cause of goiter and acquired hypothyroidism in children after iodine deficiency. In this study, clinical and laboratory findings and follow-up results of children diagnosed with HT are presented.

**Methods:**

The data of cases diagnosed with HT between 2004 and 2022 in 2 centers in Erzurum were evaluated retrospectively.

**Results:**

Of the 81 children with HT whose ages ranged from 3 to 18 years (11.24 ± 3.72), 67 (82.7%) were girls and 14 (17.3%) were boys. The most common symptoms were neck swelling (37%), fatigue (23.5%), and hair loss (23.5%). There was a family history of HT in 12 cases (9.9%). Fifty-one (63%) of the cases were in the pubertal period and 30 (37%) were in the prepubertal period. There was no goiter in 36 (44.4%) of the patients, second degree goiter in 24 (29.6%), first degree goiter in 14 (17.3%), and third degree goiter in 7 (8.7%). Twenty-two (27.2%) of the cases were euthyroid, 27 (33.3%) were subclinical hypothyroidism, 23 (28.4%) were hypothyroidism, and 9 (11.1%) were hyperthyroidism. While 18 (22.2%) of the cases were followed up without medication, 59 (72.8%) were given levothyroxine and 4 (5%) were given methimazole. The mean follow-up time was 32.1 ± 33.7 months.

**Conclusion:**

The study's findings suggest that HT is more common in girls and occurs more frequently after puberty. A personal or family history of an autoimmune disease may be a warning sign for HT. Additionally, HT should be kept in mind in the differential diagnosis of children presenting with complaints of neck swelling, fatigue, and hair loss.

**Main Points**

- Hashimoto’s thyroiditis (HT) is more common in girls and occurs after puberty.
- Children under 5 years of age, especially those with autoimmune diseases, should be evaluated for HT.
- Goiter, followed by symptoms and complaints such as fatigue and hair loss, may be clues to HT. A thyroid nodule may also accompany HT. Such cases need to be followed for thyroid cancer.

## Introduction

Hashimoto’s thyroiditis (HT) is the second most common cause of goiter and acquired hypothyroidism in children, after iodine deficiency.^1^ The disease is characterized by autoimmunity-mediated destruction of the thyroid gland. The main histopathological finding in the thyroid gland is diffuse lymphocytic infiltration accompanied by varying degrees of fibrosis.^2^ Diagnosis is based on typical ultrasound findings and the presence of antithyroid antibodies.^3^ Hashimoto’s thyroiditis can often be associated with other autoimmune diseases such as alopecia, vitiligo, celiac disease, and type 1 insulin-dependent diabetes.^3^ It has been determined that chromosomal disorders such as Down syndrome and Turner syndrome also increase the prevalence of HT.^4^

The clinical features of the disease are variable. Patients may be hyperthyroid, euthyroid, or hyperthyroid based on thyroid function tests.^2-4^ There is no previous study evaluating children diagnosed with HT in the Eastern Anatolia Region of Türkiye. The aim of this study is to examine the clinical and laboratory findings of children diagnosed with HT in the Eastern Anatolia region and to present the follow-up results.

### Material and Methods

The data of cases diagnosed with HT between 2004 and 2022 in 2 centers 2 centers in Erzurum named Atatürk University Pediatric Endocrinology Department and Health Sciences University Erzurum Regional Training and Research Hospital Pediatric Endocrinology Department were evaluated retrospectively. The diagnosis of HT was made with a heterogeneous appearance on thyroid ultrasonography and an increase in at least one of the thyroid antibodies [antithyroid peroxidase (anti-TPO) (24 Alegria® strips, product ORG 203, range 0-3000 IU/mL, cut-off 75 IU/mL, Orgentec Co., Germany) and anti-thyroglobulin (anti-Tg) (24 Alegria® strips, product ORG 202, range 0-9000 IU/mL, cut-off 150 IU/mL, Orgentec Co.)] measured in serum. Age at diagnosis, gender, symptoms, height and weight measurements, Tanner pubertal stage, degree of goiter, laboratory markers including serum free thyroxine (fT4), free triiodothyronine (fT3), thyroid-stimulating hormone (TSH), anti-Tg, and thyroglobulin (Tg) levels, thyroid ultrasonography findings, treatments given, and treatment and follow-up periods were recorded. Values >75 IU/mL for anti-TPO and >150 IU/mL for anti-Tg were considered positive results. Other autoimmune diseases such as celiac disease, type 1 diabetes mellitus (T1DM), and Addison's disease were also investigated.

Free T3 and fT4, and TSH serum levels were measured using the chemiluminescence method with the Beckman Coulter DX1800 (Beckman Coulter, Brea, USA) device during hormonal evaluation. Cases with serum TSH and fT4 levels within reference ranges (0.6-4.9 µIU/mL and 0.6-1.2 ng/dL, respectively) were regarded as euthyroid, those with only TSH >6 µIU/mL were considered subclinical hypothyroid, fT4 <0.6 ng/dL and TSH >6 µIU/mL were accepted as hypothyroid, and those with TSH <0.6 µIU/mL and fT4 >1.2 µIU/mL were considered hyperthyroid. Statistical analyses were performed on SPSS version 22 software. Descriptive statistics including mean, SD, and percentage values were performed. Distributions of longitudinal data were examined using the Kolmogorov–Smirnov test. The Wilcoxon test was applied in the analysis of non-normally distributed data and the paired *t*-test for normally distributed data. Findings were expressed as mean ± SD. *P* values <.05 were regarded as statistically significant.

This study was approved by the Health Sciences University Erzurum Regional Training and Research Hospital Ethics Committee (Approval No: 2022/06-51, Date: 16/05/2022). Written informed consent was obtained from the patients' parents who agreed to take part in the study. 

## Results

A total of 81 patients, 67 girls (82.7%) and 14 boys (17.3%) between the ages of 3 and 18, were included in the study. The average age of the patients was 11.24 ± 3.72 years. The symptoms and findings of the patients are shown in Table 1. Neck swelling was the most common symptom and finding (37%). The duration of symptoms was 3-720 days (132.6 ± 168.5). In addition to HT, 5 of the cases (6.2%) had short stature, 3 (3.7%) had T1DM, 2 (2.5%) had alopecia, 2 (2.5%) had ovarian cysts, 1 (1.2%) had thyroglossal cyst, 1 (1.2%) had autoimmune hepatitis, 1 (1.2%) had systemic lupus erythematosus, 1 (1.2%) had vitamin B12 deficiency, and 1 (1.2%) had both T1DM and juvenile idiopathic arthritis (JIA). When the families were examined, 12 (9.9%) cases had HT, 16 (19.8%) cases had goiter, 3 (3.7%) cases had thyroid nodule, 2 (2.5%) cases had Graves’ disease, 1 (1.2%) case had a non-thyroidal autoimmune disease, and 1 (1.2%) case had thyroid cancer.

Height, body weight, and body mass index (BMI) standard deviation score (SDS) values of the cases at the time of diagnosis and at the last examination are shown in Table 2.

Fifty-one (63%) of the cases were in the pubertal period and 30 (37%) were in the prepubertal period. There was no goiter in 36 (44.4%) of the patients, second-degree goiter in 24 (29.6%), first-degree goiter in 14 (17.3%), and third-degree goiter in 7 (8.7%).

Twenty-two (27.2%) of the cases were euthyroid, 27 (33.3%) were subclinical hypothyroidism, 23 (28.4%) were hypothyroidism, and 9 (11.1%) were hyperthyroidism.

The mean serum fT4 value was 0.9 ± 0.5 ng/dL (N: 0.6-1.2), mean TSH 25.2 ± 34.4 mIU/L (N: 0.6-4.9), Tg 57.9 ± 99.2 ng/mL (N: 1.6-60), anti-Tg 761.0 ± 767.9 IU/mL (N: 0-150), and anti-TPO 4345.7 ± 33.824.0 IU/mL (N: 0-75). It was determined that both antibodies were positive in 44 (54.3%) of the cases, only anti-TPO in 27 (33.3%), and only anti-Tg positive in 8 (9.87%). The distribution of positive thyroid autoantibodies according to thyroid functions is shown in Table 3. These antibodies were most frequently positive in the cases with hypothyroidism and subclinical hypothyroidism.

While no correlation was detected between serum anti-TPO levels and TSH, fT4, or fT3 levels (*P* > .05), a positive correlation was found between anti-Tg levels and TSH (*r* = 0.251 and *P* = .03).

On thyroid ultrasonography, thyroid gland sizes were normal in 53 (68.8%) cases, large in 20 (26%) cases, and small in 4 (5.2%) cases. Ectopic thyroid tissue was also detected in 1 case. Thyroid tissue was heterogeneous in appearance in 74 (91.4%) cases. Pseudonodules were detected in 34 (42%) cases and thyroid nodules were detected in 18 (22.2%) cases. Fine needle biopsy aspiration was performed in 2 (2.7%) cases with thyroid nodules, and histopathological findings were reported as benign.

While 18 (22.2%) of the cases were followed up without medication, 59 (72.8%) were given levothyroxine and 4 (5%) were given methimazole. All of the cases presenting with hyperthyroidism became euthyroid during follow-up. The average follow-up period was 32.1 ± 33.7 months. At the last follow-up, 52 cases (64.2%) were using levothyroxine and 20 cases (24.7%) were being followed up without medication. Follow-up of 9 (11.1%) cases was terminated.

## Discussion

Hashimoto’s thyroiditis is the most common cause of all thyroid diseases and acquired hypothyroidism with or without goiter in children and adolescents.^5^ The prevalence of HT in children is 1.2-3%, and it is 2-4 times more common in girls.^2^ The disease usually peaks in post-pubertal girls. In studies from Türkiye, the female/male ratio varies between 6.4/1 and 2.8/1.^6-8^ Tunç et al^7^ reported that 68% of the cases occurred in the pubertal period. In our study, the female/male ratio was 4.8/1 and 63% of our cases were in the pubertal period. These findings suggest that the situation in our region is compatible with other data from Türkiye (Table 4).

In the literature, the mean age of HT varies between 11.1 ± 3.0 and 12.2 ± 2.8 years.^7-9^ The mean age of the patients in our study was 11.24 ± 3.72 years, a finding consistent with previous literature. HT is rare below the age of 5. However, cases diagnosed in infancy have also been reported.^10^ There were 4 cases under the age of 5 in our study. While 1 of them had no complaints, 1 had short stature and the others had autoimmune diseases. While one of these cases had combined T1DM and JIA, autoimmune hepatitis was detected in the other. These findings suggest that HT can also be seen in children under 5 years of age and that young children with autoimmune diseases should also be evaluated for HT. In one study, T1DM and celiac disease were identified as the most common autoimmune diseases accompanying HT. That study also identified vitiligo as the most common dermatological disease accompanying HT.^9^ In our study, it was found that the most common autoimmune disease accompanying HT was T1DM, and the most common dermatological disease was alopecia.

Hashimoto’s thyroiditis is known to exhibit a genetic and familial disposition. A Korean study reported that the risk of HT in individuals with a history of HT in first-degree relatives was 6.5 times higher than in individuals without first-degree relatives with HT.^11^ Previous studies reported that 23-46% of children diagnosed with HT had a family history of HT or autoimmune disease.^7^ In our study, 9.9% of the families had a history of HT. Detailed history revealed a family history of thyroid disease in 42% of cases. This percentage value is consistent with other studies in Türkiye (Table 4). These findings suggest that patients should be evaluated for HT in the presence of HT or autoimmune disease in the family.

Hashimoto’s thyroiditis patients are usually asymptomatic at the time of diagnosis. The most frequently reported clinical findings in symptomatic patients are goiter and growth failure.^12^ Consistent with the literature, we found that the most common complaint was goiter, followed by fatigue and hair loss. Growth retardation was detected in 8.6% of our patients, while 13.5% were asymptomatic.

While the majority of children with HT are reported to be euthyroid at the time of diagnosis, it has been shown that hypothyroidism occurs in the remaining cases, followed by subclinical hypothyroidism and hyperthyroidism in decreasing order of prevalence.^4,6-8,13^ Consistent with the literature, the least common clinical picture in our study was hyperthyroidism, while the most common clinical picture was subclinical hypothyroidism. This may be attributed to the small number of our patients and the late presentation of our cases. In our study, the hyperthyroidism rate was 11.1%, which is consistent with Demirbilek et al.^8^ Interestingly, all of the cases presenting with hyperthyroidism were girls.

It has been shown that children with HT have a higher prevalence of developing papillary thyroid cancer (PTC) compared to the normal population. Papillary thyroid cancer has been reported to develop in 3.07% of patients with HT.^14^ In a study conducted in Korea, the prevalence of thyroid nodules in children and adolescents with HT was determined as 22.4% and the malignancy rate was 7.9%.^15^ Lee et al^16^ determined the prevalence of nodules in HT patients as 18.8% and the risk of malignancy as 22.5%. Studies conducted in Türkiye report that malignancy rates are between 0.62% and 1.3%.^1,8^ The prevalence of thyroid nodules in this study was 22.2%, consistent with previous reports from Türkiye. However, no malignancy was detected in any patient with thyroid nodules. This may be due to the low number of cases and insufficient follow-up period.

The second most common condition detected according to thyroid function tests in children with HT is hypothyroidism. Hypothyroid cases need to be given thyroid hormone replacement therapy. Tuhan et al^6^ determined the L-thyroxine treatment rate in children with HT as 71.3%.^6^ In our study, the L-thyroxine treatment rate was 72.8%, which is similar to the rate reported by Tuhan et al but higher than the rate reported by Dündar et al and Tunç et al.^7,13^ This may be due to the small number of euthyroid patients in our study.

Our study has some limitations. We did not measure urine iodine levels. Some of the hypothyroidism patients may have iodine deficiency, and therefore the rate of hypothyroidism patients may have been high in this study.

The findings of this study indicate that HT is more common in girls after puberty. Children under 5 years of age, especially those with autoimmune diseases, should be evaluated for HT. It should be kept in mind that HT may develop in children with a family history of HT or autoimmune disease. Goiter, followed by symptoms and complaints such as fatigue and hair loss, may be clues to HT. A thyroid nodule may also accompany HT. Such cases need to be followed for thyroid cancer. In cases where hypothyroidism and hyperthyroidism develop, appropriate treatments should be given.

## Data Availability Statement

The data that support the findings of this study are available on request from the corresponding author.

## Figures and Tables

**Table 1.** Presentation Findings of a Total of 81 Cases

	n	(%)
Neck swelling	30	37
Hair loss	19	23.5
Fatigue	19	23.5
Irritability	14	17.3
Excessive sleep	10	12.3
Cold intolerance	10	12.3
Palpitation	9	11.1
Sweating	9	11.1
Poor school performance	8	9.9
Constipation	7	8.6
Interruption of growth	7	8.6
Weight gain	7	8.6
Dry skin	7	8.6
Headache	6	7.4
Pruritus	2	2.5
Abdominal swelling	1	1.2
Gynecomastia	1	1.2

**Table 2.** Height, Body Weight, and Body Mass Index Standard Deviation Score Values of the Cases at the Time of Diagnosis and at the Last Examination

Parameter	At Time of Diagnosis	At the Last Examination	*P*
Height SDS	−0.57 ± 1.2	−0.33 ± 1.09	.001
Body weight SDS	−0.32 ± 1.3	−0.28 ± 1.25	.001
Body mass index SDS	0.05 ± 1.1	0.45 ± 3.3	.383

SDS, standard deviation score.

**Table 3.** The Distribution of Positive Thyroid Autoantibodies According to Thyroid Functions in 81 Cases Diagnosed with Hashimoto Thyroiditis

	Euthyroid, n (%)	Subclinical Hypothyroidism, n (%)	Hypothyroidism, n (%)	Hyperthyroidism, n (%)
Anti-TPO	18 (22.2%)	26 (32.0%)	20 (24.6%)	7 (8.6%)
Anti-Tg	10 (12.3%)	18 (22.2%)	18 (22.2%)	6 (7.4%)

anti-Tg, anti-thyroglobulin; anti-TPO, antithyroid peroxidase.

**Table 4.** The Clinical and Laboratory Characteristics of Children with Hashimoto’s Thyroiditis in Studies from Türkiye

	Demirbilek *H*	Dündar *B*	Tunç *S*	Tuhan *H*	The Present Study
Case number	162	78	108	80	81
Mean age	11.4 ± 2.97	12.2 ± 0.31	12.2 ± 2.8	10.6 ± 3.4	11.24 ± 3.72
Most frequent accompanying autoimmune disease	T1DM	None	Celiac disease, T1DM		T1DM
Family history of thyroid disease	41.1%	39%	59%	47.5%	42%
Female/male ratio	6.4/1	6/1	2.8/1	4.3/1	4.8/1
Puberty		71.7%	68%	72.5%	63%
Presentation finding	Goiter (54.9%)	Irritability, excessive sweating	Swelling in the neck, weight gain, fatigue	Swelling in the neck, weight gain, fatigue	Swelling in the neck, hair loss, fatigue
Goiter at physical examination	90.7%	80.8%	43.5%	46.2%	55.6%
Thyroid functions	43.2 euthyroid, 24.1% subclinical hypothyroidism, 21% hypothyroid 11.7% hyperthyroid	62.8% euthyroid, 21.8% subclinical hypothyroidism, 11.7% hypothyroid, 2.6% hyperthyroid	44.4% euthyroid, 35% subclinical hypothyroidism, 16.6% hypothyroid, 3.7% hyperthyroid	53.8% euthyroid, 28.7% subclinical hypothyroidism, 17.5% hypothyroid,	27.2% euthyroid, 33.3%subclinical hypothyroidism, 28.4% hypothyroid, 11.1% hyperthyroid
Thyroid USG findings	Heterogeneity 92.9%, nodule 19.2%	Heterogeneity 66.2%, nodule 7%	Heterogeneity 88%, pseudonodular appearance 83%, nodule 17%	Pseudonodular appearance 46.3%, nodule 7.5%	Heterogeneity % 91.4%, pseudonodular appearance 42%, nodule 22.2%
Anti-Tg positivity rate			87%	82.5%	64.2%
Anti-TPO positivity rate		66.7%	95%	100%	87.7%
Malignancy rate	0.62%	0%			0%
Treatment rate		64.1%	43.5%	71.3%	72.8%

anti-Tg; anti-thyroglobulin; anti-TPO, antithyroid peroxidase; T1DM, type 1 diabetes mellitus; USG, ultrasonography.
